# Dysphagia as an Unusual Presentation of Myeloma

**DOI:** 10.1155/2018/6910624

**Published:** 2018-12-13

**Authors:** A. Raissi, Z. Chahbi, M. Zyani, Y. Darouassi

**Affiliations:** ^1^Clinical Hematology, Avicenna Military Hospital, Marrakech, Morocco; ^2^Internal Medicine, Avicenna Military Hospital, Marrakech, Morocco; ^3^Otorhinolaryngology Department, Avicenna Military Hospital, Marrakech, Morocco

## Abstract

Multiple myeloma is a plasma cell dyscrasis characterized by mature B cells proliferation in the bone marrow. In rare cases, the disease can present as an extramedullary location, making diagnosis and management more challenging. Here, we describe a rare case of tongue extramedullary myeloma and discuss diagnostic, prognostic, and therapeutic issues.

## 1. Introduction

Multiple myeloma (MM) is a plasma cell dyscrasis characterized by mature B cells proliferation in the bone marrow. Hematogenous spread of plasma cells can occur in up to 30% of myeloma patients during the disease course, generating soft tissue plasmacytoma (STP) [1]. In rare cases, extramedullary disease may be the first symptom, making diagnosis and management more challenging.

Here, we describe an atypical case of tongue base STP revealing symptomatic MM and discuss diagnostic, prognostic, and therapeutic issues.

## 2. Case Report

A sixty-one-year old male, in otherwise good health, was referred to otorhinolaryngology emergency for a one-year history of progressive dysphagia and recent dysphonia and dyspnea.

Intraoral clinical examination revealed an enormous mass (80 × 55 mm in size) filling the oropharynx and surpassing the anterior pillars. The lesion was firm and tender and showed no fluctuation. Cervical lymph nodes were not palpable. The remaining clinical examination revealed pain in the right lower limb.

Upon arrival in the emergency room, a rescue tracheotomy was performed.

**Figure 1:**
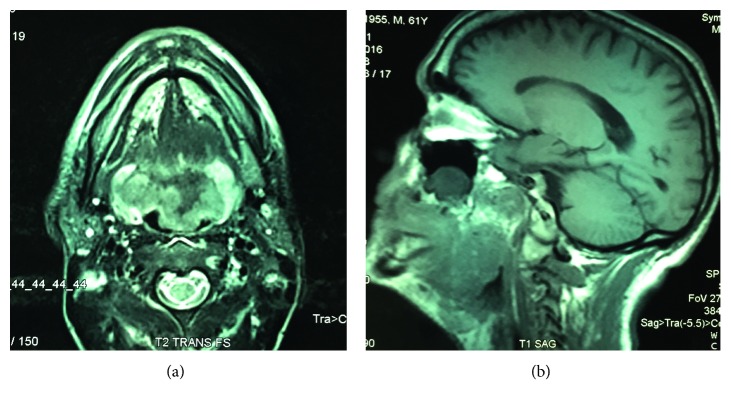
Sagittal and transversal MRI sections showing a voluminous process of the posterior tongue infiltrating the uvula and tonsils.

Magnetic resonance imaging (MRI) showed a voluminous process occupying and infiltrating the entire posterior tongue. The lesion was heterogeneous on T1 and T2 tissue signal. Imaging was enhanced after injection of GADO (measuring 75 × 55 × 39 mm along major axes). This process infiltrated the uvula and the tonsils (Figure 1). No associated cervical lymph nodes were found.

**Figure 2:**
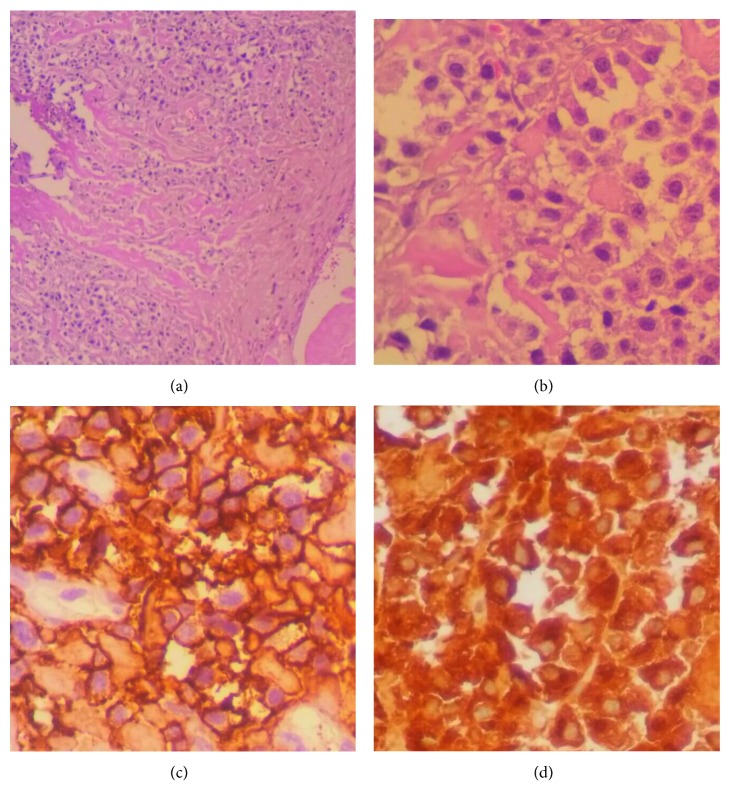
Tongue base biopsies showing massive infiltration with plasmacytic differentiation cells, comforted by the positivity of CD138 and lambda chains.

Biopsy of the tongue showed a squamous mucosa whose chorion was massively infiltrated by tumoral proliferation with distinct plasmacytic differentiation. Immunohistochemistry showed positivity of CD138 and monoclonal expression of Lambda chains (Figure 2).

**Figure 3:**
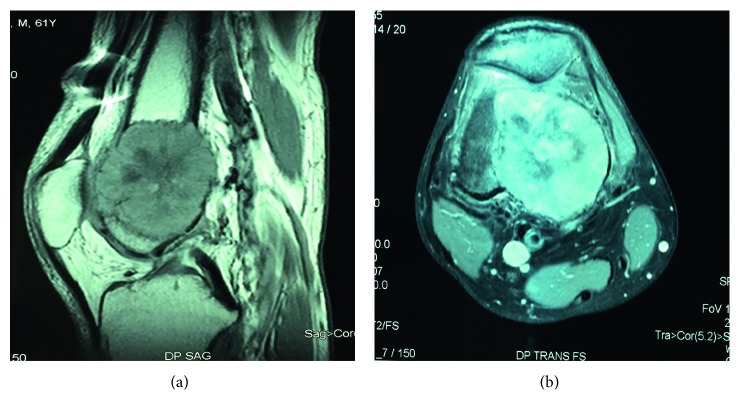
Sagittal and transversal MRI sections of the right knee showing plasmacytoma of the lower right femur metaphysis blowing the inner cortical.

To screen for other locations, we performed whole-body MRI which demonstrated a lesional process occupying the lower metaphysis of the right femur. The lesion was rounded with polylobed contours and had an intermediate signal in T1 with a central zone in hypersignal. It destroyed the inner cortical and measured 61 × 47 × 47 mm according to major axes (Figure 3). Spine MRI did not demonstrate signal abnormalities of the vertebral bodies or the spinal cord.

Laboratory workup showed a 7.5 g/dl normocytic anemia and accelerated sedimentation rate. Calcemia and renal function were of normal range. There was no clear monoclonal peak in serum electrophoresis. The serum and urinary immunofixation revealed lambda chains confirmed with nephelemetric free light chains dosage. Beta2 microglobulin was slightly elevated.

**Figure 4:**
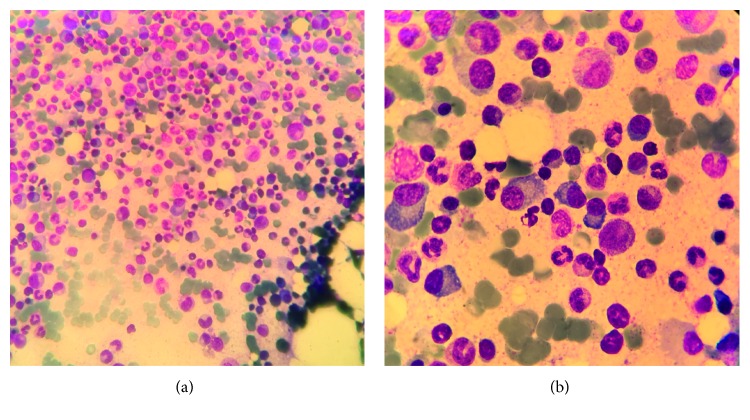
Bone marrow smears showing infiltration with dystrophic plasma cells: (a) 40x magnification; (b) 100x magnification.

Bone marrow aspiration showed 12% dystrophic plasma cell medullary infiltration, with presence of some plasma cells clusters (Figure 4). Screening of prognostic genetic abnormalities by conventional cytogenetics and fluorescent in situ hybridisation (FISH) was negative.

The patient received VTD induction therapy according to local guidelines (bortezomib: 1.3 mg/m^2^ and dexamethasone: 40 mg both on days 1, 4, 8, and 11 and thalidomide 100 mg daily in a 21-day schedule).

After 4 courses, the patient was in complete remission (CR) according to International Myeloma Working Group (IMWG) criteria [1]. He then received autologous stem cell transplantation (ASCT) after conditioning with melphalan 200 mg/m^2^. Two additional VTD cycles were given posttransplant as consolidation. Patient then started maintenance treatment with bortezomib at a dose of 1.3 mg/m^2^ every 2 weeks for a planned total duration of 2 years.

Overall, treatment was well tolerated with no grade 3 or more side effects.

One year after ASCT, the patient is still on bortezomib maintenance, with good tolerance and no sign of relapse.

## 3. Discussion

The presence of STP in a MM patient defines extramedullary myeloma (EMM). However, this term can be more global including all extramedullary disease (i. e., solitary plasmacytoma of the bone and plasma cell leukemia). But experts tend to recommend using this term in MM with only STP [2, 3].

The incidence of extramedullary myeloma is not well known, due to the lack of studies specifically addressing the question. It varies widely between series, ranging from 6 to 30%, mainly due to the nonharmonization of the definition of EMM and the inclusion, in some series, of other diagnostic or therapeutic criteria (i. e., IgD myeloma, younger age, response to pomalidomide, and de novo or relapsed MM) [4–6].

Using the definition restricted to the presence of STP only, the incidence of EMM at diagnosis of MM ranges from 6 to 8% based on whole-body MRI or 18-fluorodesoxyglucose positron emission tomography (PET-FDG) imaging [5, 7, 8].

However, this low incidence may not be completely true, because some autopsy series showed the presence of plasma cells outside the bone marrow at a much higher level than that found by radiology [9].

A higher incidence of extramedullary relapse was reported in patients who underwent allogeneic stem cell transplantation and those treated with newer antimyeloma agents (i. e., bortezomib and thalidomide) [10–14]. Nevertheless, younger age of stem cell recipients always presenting aggressive disease characteristics on the one hand and the prolongation of survival in myeloma patients treated with newer agents on the other hand may explain this high frequency of EMM at relapse.

Several mechanisms of plasma cell extramedullary dissemination were described (Tables 1 and 2) [9].

Thus, all tissues can be affected with some localization more frequent than others (skin, liver, breast, kidney, lymph nodes, and central nervous system (CNS)). The location at the tongue is very rare and was limited to a few reported cases [15–18]. This location is even rarer when it is associated to symptomatic MM.

Diagnosis is usually made by histopathological studies. To minimize the risk of false-negative results, it is important to perform deep biopsies, since 80% of STP develop from the submucosa [19].

Plasma cells from STP are different from those from bone-related plasmacytomas; they usually show immature and plasmablastic features [9].

Patients with EMM tend to have less hemoglobin and platelets levels, elevated serum LDH, and more high-risk cytogenetic abnormalities [2, 20].

Compared to other myelomas, extramedullary disease is associated with a poor prognosis both at diagnosis and relapse (short-term PFS and OS), with some localizations associated with an even worse prognosis (liver, lung, and muscle). This fact persists despite the use of new drugs in the management of MM [21].

There is no prospective data regarding the management of EMM patient. Expert recommendations stipulate that de novo patients should be treated intensively [2].

Fit patients should benefit from a triplet induction regimen followed by autologous stem cell transplantation (ASCT) and maintenance therapy. Some authors have even proposed routine use of tandem ASCT.

Given the atypical presentation of the disease, the high risk of relapse, and the relatively young age of the patient (despite the absence of poor prognostic genetic abnormalities), we put the patient on maintenance treatment with bortezomib at a dose of 1.3 mg/m^2^ every 2 weeks for a planned total duration of 2 years.

Patients who are not eligible for ASCT should receive treatment with bortezomib or continuous lenalidomid (VMP, Rd).

CNS location is a particularly rare and aggressive complication. It must be treated by intrathecal (IT) chemotherapy, radiation, and combination of antimyeloma drugs that cross the blood-brain barrier (high-dose dexamethasone, imids, and bendamustine) [2]. In this setting, Mussetti et al. reported sustained CNS remission after pomalidomide and dexamethasone treatment [22]. Nahi et al. have also reported good results after treatment with combination of thalidomide, bendamustine, and radiotherapy [23].

Treatment with vemurafenib has shown good results in a BRAF V600E-mutated EMM patient [24]. Immune therapy with chimeric antigen receptor T-cells also showed promising results in a limited number of patients [25].

In relapsed patients, previous received therapies, type of relapse, and remission duration in association of patient general condition should be considered in decision-making. Pomalidomid have shown good results in relapsed EMM [6].

Novel approaches in the treatment of relapsed/refractory myeloma are ongoing, especially the use of monoclonal anti-CD38 antibody daratumomab or anti-SLAMF7 elotuzumab in combination with bortezomib or lenalidomide and dexamethasone.

## 4. Conclusion

Our case highlights the importance of identifying extramedullary disease at diagnosis of MM. Prognosis of EMM is poor in both de novo and relapsed/refractory situations. Thus, clinicians should screen for extramedullary disease using either whole-body MRI or PET-FDG imaging. Histological confirmation of suspected locations should be systematically performed. A better understanding of the pathophysiology of EMM may determine a unique genetic signature that can be used in the future to develop new-targeted therapies.

## Figures and Tables

**Table 1:** Extramedullary myeloma: growth and location, adapted from [9].

Mechanism	Location
Local growth	Soft-tissue masses arising from focal bone involvement (vertebrae, ribs, sternum, and skull)
Hematogenous spread	Single or multiple large subcutaneous tumors Multiple nodules (skin, liver, breast, and kidney) Lymph nodes CNS
Triggered by invasive surgical procedures	Surgical scars (laparotomy and catheter insertions) Bone surgery and/or fractures (extensive local spread)

**Table 2:** Possible mechanism of extramedullary spread, adapted from [9].

Mechanism	Factors involved
Decreased expression of adhesion molecules	VLA-4, CD44, P-selectin
Downregulation of chemokine receptors	CCR1, CCR2, CXCR4
Downregulation of tetraspanins expression	CD81, CD82
Increased heparanase-1 expression	Angiogenic and metastasic potential
Angiogenesis	VEGF, MMP-9, angiopoetin-1, CD31, endoglin
Activation pathway	Mutations in alternative or classical NF-kB

MMP-9: matrix metalloproteinase-9; NF-kB: nuclear factor kB; VEGF: vascular endothelial growth factor.
